# A Systematic Review of the Therapeutic Outcome of Mucormycosis

**DOI:** 10.1093/ofid/ofad704

**Published:** 2023-12-30

**Authors:** L Shamithra M Sigera, David W Denning

**Affiliations:** Manchester Fungal Infection Group, Core Technology Facility, University of Manchester, Manchester Academic Health Science Centre, Manchester, UK; Manchester Fungal Infection Group, Core Technology Facility, University of Manchester, Manchester Academic Health Science Centre, Manchester, UK

**Keywords:** *Absidia*, *Apophysomyces*, *Lichtheimia*, *Mucor*, *Rhizomucor*, *Rhizopus*

## Abstract

**Background:**

Mucormycosis is a potentially lethal mycosis. We reviewed peer-reviewed publications on mucormycosis to assess therapeutic outcomes.

**Methods:**

A systematic literature search using the Ovid MEDLINE and EMBASE databases identified manuscripts describing human mucormycosis diagnosed according to European Organization for Research and Treatment of Cancer and the Mycoses Study Group criteria with therapeutic outcomes published from 2000 to 2022.

**Results:**

In 126 articles, 10 335 patients were described, most from Asia (n = 6632, 66%). Diabetes was the most frequent underlying disease (n = 6188, 60%); 222 (2.1%) patients had no underlying diseases. The dominant clinical form was rhino-orbitocerebral (n = 7159, 69.3%), followed by pulmonary (n = 1062, 10.3%). Of 5364 patients with outcome data, amphotericin B monotherapy (n = 3749, mortality 31.5%) was most frequent, followed by amphotericin B + azole (n = 843, mortality 6.6%; *P* < .0001), amphotericin B followed by azole (n = 357, mortality 13.7%; *P* < .0001), posaconazole only (n = 250, mortality 17.2%; *P* < .0001), and isavuconazole only (n = 65, mortality 24.6%; *P* = .24). Duration and dose of antifungals varied widely. Documented outcomes from surgical resections in 149 patients found that 47 of 125 died (37.6%), compared with 16 of 24 (66.7%) patients who did not undergo surgery (*P* = .008).

**Conclusions:**

Mucormycosis is more frequently reported in Asia than in Europe and is often linked to diabetes. Antifungal therapy, usually with surgery, is frequently effective for mucormycosis.

## Teaser

Untreated mucormycosis has a 100% mortality, reduced to ~32% with amphotericin B, ~14% with amphotericin B followed by azole (possible survivor bias), 17-25% with posaconazole or isavuconazole alone, or ~7% in combination with amphotericin B. Surgical resection(s) reduces mortality from ~67% to ~38%.

Mucormycosis is a potentially lethal mycosis caused by filamentous fungi of the subphylum Mucoromycotina [1, 2]. Fungi in the subphylum Mucormycotina are saprophytic fungi that are ubiquitous in nature [2, 3]. They are frequently isolated from decaying organic material, soil, and compost piles [3].

Human infection follows the inhalation of fungal spores, traumatic inoculation, and consumption of contaminated food [4]. Mucormycosis is an aggressive opportunistic fungal infection that is difficult to manage [4]. The characteristic nature of angioinvasion can lead to tissue necrosis, vascular thrombosis, and dissemination [4]. Since the first reported case of human mucormycosis by Paltauf in 1885, this potentially lethal infection gained great attention during the coronavirus disease 2019 (COVID-19) pandemic, reaching almost epidemic proportions in India [5].

Mucorales is second in frequency to *Aspergillus* spp. as a cause of invasive fungal infections in transplanted recipients and patients with malignancies [6]. The perceived increased number of mucormycosis cases globally may be attributable to the increasing prevalence of diabetes mellitus, especially in low- and middle-income countries, and use of new immune-modulating agents against autoimmune diseases, cancer, and transplantation [3]. The enhanced frequency of case recognition may also be attributed to improved awareness, increased expertise, and improved competence in the diagnosis [3].

A modern comprehensive literature review on the outcomes of mucormycosis is lacking. We therefore reviewed cases series and studies of mucormycosis published from 2000 to 2022 to deliver an enhanced understanding of case distribution, diagnosis, and management across the world.

## METHODS

A systematic literature search was performed using the Ovid, MEDLINE, and EMBASE databases to identify manuscripts describing human mucormycosis according to European Organization for Research and Treatment of Cancer and the Mycoses Study Group (EORTC/MSG) criteria with therapeutic outcomes published from 2000 to December 2022. The following search terms were used during the identification of the articles: (mucormycosis OR zygomycetes OR *Rhizopus* spp, OR *Mucor* spp OR *Lichtheimia* spp OR *Absidia* OR *Mycocladus* OR *Rhizomucor* spp, OR *Cunninghamella* spp, OR *Apophysomyces* spp, OR *Saksenaea* spp) AND (rhinocerebral mucormycosis OR rhino orbital mucormycosis OR gastrointestinal mucormycosis OR cutaneous mucormycosis OR disseminated mucormycosis OR renal mucormycosis). Single case reports, case series of <10 cases, reviews, editorials, letters, conference abstracts, and animal studies were excluded from the search. Articles were screened to identify human cases of mucormycosis diagnosed according to EORTC/MSG criteria, which required a diagnosis confirmed by histopathology or direct microscopy and/or culture from an invasive specimen in patients at risk with a characteristic clinical syndrome (and therefore included both confirmed and probable cases). Articles that provided antifungal names, duration (>1 day), and outcomes (death or cure) were selected to obtain data on management. Cases series in languages other than English were read, and if the abstract justified translation, a translation was obtained.

### Database Development

All data were extracted by author L.S.M.S. into a database using Excel, with specific input on problematic papers provided by author D.W.D. The categorical variables described included gender, predisposing factors, diagnostic method, site of infection, antifungal treatment, surgical treatment (not diagnostic biopsy alone), and outcome. The continuous variables described were year of publication, year of diagnosis, patient age, antifungal dose, and/or duration. Antifungal therapies were grouped according to the predominant therapy provided. Antifungal therapy given for at least 1 day was considered positive for antifungal therapy. Combined therapy of amphotericin B with either posaconazole or isavuconazole was categorized as amphotericin B plus azole therapy. Amphotericin B followed by either posaconazole or isavuconazole was termed amphotericin B then azole therapy. Combined therapy of amphotericin B and any echinocandin was considered amphotericin B plus echinocandin therapy. Therapeutic outcome was analyzed by individual patient and by cohort (depending on the paper) and then merged.

### Statistical Analysis

All the collected data were analyzed manually. Descriptive analyses were used to summarize the demographic and clinical features of the patients in the different series. No attempt was made to statistically compare these data. Outcome data were compared using the chi-square statistic with amphotericin B alone as the standard therapy.

## RESULTS

**Figure 1.**
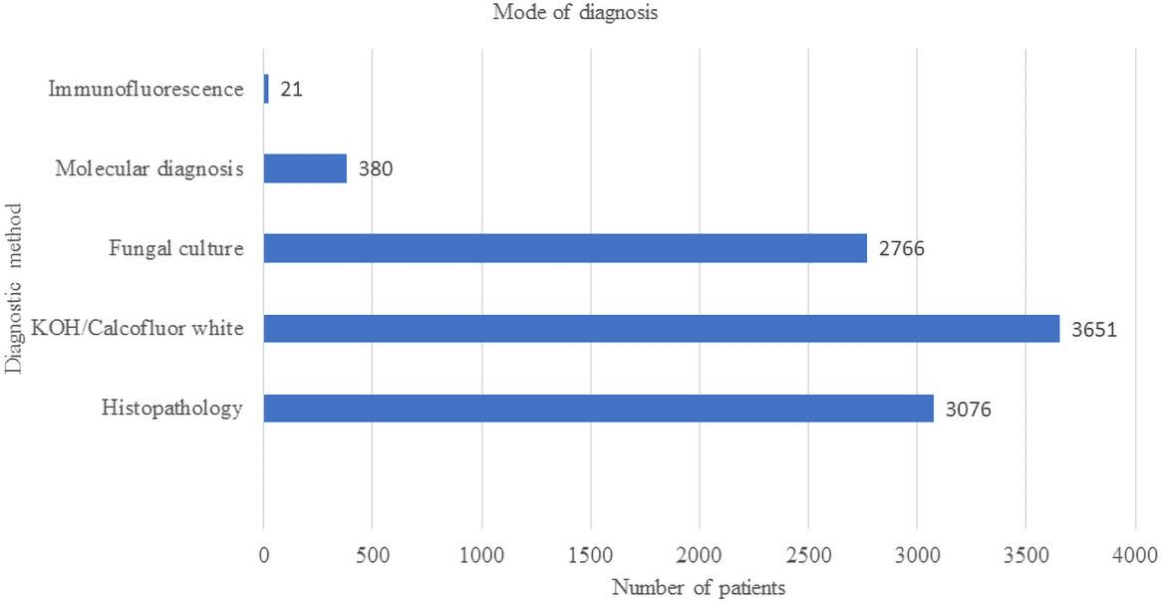
The mode of diagnosis of 10 335 cases of reported cases of mucormycosis. Abbreviation: KOH, potassium hydoxide.

The primary database searches identified 2322 articles (Supplementary Data 1), from which 2196 articles, either duplicates or articles that did not satisfy inclusion criteria, were excluded. One hundred twenty-six articles that fulfilled the inclusion criteria were included in the final analysis regarding demographics (Supplementary Data 2, Supplementary Figure 1). Publications yielded clinical information from 1971 to 2022. Underlying diseases, geography, mode of diagnosis, and body site of infection were extracted from 126 articles. Satisfactory data on antifungal management and outcomes were available for 5364 patients. Individual patient treatment data on 150 patients were available in 11 articles, and group data on 5214 patients were provided in 23 articles (Figure 1; Supplementary Data 3). Information on surgical management was obtained from 149 patients whose data was provided individually in 11 articles (Supplementary Data 4).

### Patients’ Demographic Characteristics

A total of 10 335 patients were described in the selected journal articles. The mean age range was 12.6–62 years, and the median age range was 8–91 years. Males outnumbered females and accounted for 66% of the population. The majority of the patients were from Asia (n = 6632, 66%) (Supplementary Figure 2), followed by North America (n = 1686, 17%), Europe (n = 1119, 11%), the Middle East (n = 645, 6%), and Australia (n = 29) (Supplementary Figure 1). India has published most of the Asian cases (n = 6113, 60%), followed by China (n = 219), Taiwan (n = 204), and South Korea (n = 96). A few published studies describe patients from multiple countries (n = 224) depicted as a separate category.

### Underlying Disease Conditions and Predisposing Factors

Diabetes was the most common underlying condition related to mucormycosis (n = 6188, 60%), with 469 (4.5%) reported to have diabetic ketoacidosis (Table 1). The next most common underlying condition was COVID-19 at 1732 (17%), of whom 403 received remdesivir. Hematological disease was the underlying condition in 1697 patients (16.4%; AML/ALL [n = 1626], aplastic anemia [n = 38], non-Hodgkin lymphoma [n = 16], or myelodysplastic syndrome [n = 14]). Neutropenia was documented in 674 (6.5%) patients. There were 564 patients who had a history of HSCT (including 175 with graft-vs-host disease), 356 recipients of a solid organ transplant, and 16 cases who had undergone re-transplantation. Trauma or burn was reported in 2.3% and 0.2% of patients, respectively. Trauma (n = 241), surgery (n = 37), and burns (n = 21) were observed among patients with cutaneous mucormycosis. Only 176 (1.7%) patients had solid tumours. It was observed that 5491 (53%) patients were on immunosuppressive treatment, most often corticosteroids (n = 4669, 45%). Other types of immunosuppressive therapy included immunosuppressive therapy not specified (n = 515, 5%), chemotherapy (n = 284, 2.7%), or tocilizumab/bevacizumab (n = 123). Remarkably, 222 (2.1%) patients had no obvious predisposing factors.

**Table 1.** Underlying Disease Conditions and Predisposing Factors

Underlying Conditions	Subcategory	Number	Percentage
Diabetes	…	**6188**	**60**
…	Diabetic ketoacidosis	469	4.5
COVID-19	…	**1732**	**17**
Hematological diseases	…	**1697**	**16**.**41**
…	AML/ALL	1626	16
…	Aplastic anemia	38	0.36
…	NHL	16	0.15
…	Myelodysplastic syndrome	14	0.13
Hypertension	…	856	8.28
HSCT	…	564	5.45
Solid organ transplantation	…	356	3.44
GVHD^a^	…	189	1.82
Solid tumors	…	176	1.7
Autoimmune disease	…	20	0.19
Respiratory diseases (interstitial lung disease n = 1, asthma n = 5, COPD = 56, other respiratory disease n = 15)	…	77	0.74
Chronic renal disease	…	396	3.83
Dialysis	…	74	0.71
Liver disease	…	74	0.71
Others including primary immunodeficiency (n = 3), chronic sinusitis (n = 22), CMV infection (n = 25), HIV (n = 1), pregnancy (n = 1)	…	52	0.50
Predisposing factor			
Corticosteroid therapy	…	4669	45.17
Neutropenia	…	674	6.52
Immunosuppressive therapy unspecified		515	4.98
Chemotherapy	…	284	2.74
Trauma	…	241	2.33
Tocilizumab/bevacizumab	…	123	1.19
Surgery	…	37	0.35
ICU care	…	27	0.26
Malnutrition	…	24	0.23
Burns	…	21	0.20
Others including smoking (n = 18) and deferoxamine (n = 3)	…	21	0.20
Intravenous drug addiction	…	8	0.077
No obvious predisposing factors	…	222	2.14

The bold values represents the total and % for that category. Under it is a subcategory.

Abbreviations: ALL, acute lymphoblastic leukemia; AML, acute myeloid leukemia; CMV, cytomegalovirus infection; COPD, chronic obstructive pulmonary disease; COVID-19, coronavirus disease 2019; GVHD, graft-vs-host disease; HSCT, hematopoietic stem cell transplantation; ICU, intensive care unit; NHL, non-Hodgkin lymphoma.

^a^Includes 14 GVHD cases after solid organ transplant.

### Antifungal Prophylaxis

There were 1001 patients with a history of antifungal prophylaxis (Supplementary Figure 3). Voriconazole was the most frequently prescribed antifungal (n = 323), followed by fluconazole (n = 245), amphotericin B (n = 131), echinocandin (n = 139), posaconazole (n = 89), and itraconazole (n = 47). However, the duration of antifungal prophylaxis before diagnosis of mucormycosis was not clearly documented.

### The Clinical Form of Mucormycosis

The dominant clinical form of mucormycosis was rhino-orbital cerebral mucormycosis (ROCM; 7159, 69.2%), followed by pulmonary (n = 1062, 10.3%), disseminated (n = 605, 5.8%), cutaneous (n = 433, 4.1%), and gastrointestinal localization (n = 149, 1.4%) (Supplementary Figure 4). Renal mucormycosis was documented in 70 (0.7%) patients.

### Diagnostic Methods

The majority of patients were diagnosed with direct microscopy using either potassium hydroxide or Calcofluor white (n = 3651). Both histopathology (n = 3076) and culture (n = 2766) were also commonly performed for the diagnosis (Figure 1). Molecular methods (n = 380) and immunofluorescence (presumptively on tissue; n = 21) were occasionally employed but infrequently. Although molecular methods were still infrequently used, these methods were applied in all regions including Australia, China, Europe, India, the Middle East, and North America. Whether the molecular diagnostic method was used for primary diagnosis or pathogen identification was not clear.

### Management

#### Antifungal Therapy

A total of 4974 patients received amphotericin B at some stage of treatment either as monotherapy or combination therapy. Amphotericin B deoxycholate was given to 826 patients as either as monotherapy or combination therapy. The remainder of the patients were treated with lipid formulations of amphotericin B either as monotherapy or combination therapy (amphotericin B colloidal dispersion [n = 4], amphotericin B lipid complex [n = 23], liposomal amphotericin B [n = 4038]). The type of amphotericin B preparation was not mentioned in 83 patients.

Amphotericin B was the only medical therapy in 3749 (70%) patients (Supplementary Figure 5). Amphotericin B deoxycholate was given to 791 patients as monotherapy. The type of amphotericin B was not specified in 6 patients, and the remainder were treated with lipid formulations of amphotericin B monotherapy (amphotericin B colloidal dispersion [n = 4], amphotericin B lipid complex [n = 18], liposomal amphotericin B [n = 2930]). By body site, it was used for ROCM (n = 3195), multiple infection sites (n = 499), pulmonary (n = 22), disseminated (n = 14), skin and soft tissue (n = 8), renal (n = 7), and sinus (n = 4) mucormycosis cases.

The second most common antifungal management observed was amphotericin B and azole combination (n = 843, 15.7%), and this combination was used for management of ROCM (n = 750), multiple sites (n = 92), and pulmonary (n = 1) cases. Other antifungal management choices were amphotericin B followed by azole (n = 357, 6.7%), posaconazole alone (n = 250, 4.7%), isavuconazole alone (n = 65, 1.2%), amphotericin B and echinocandins (n = 25), and an echinocandin alone (n = 1). The dose and duration of administered antifungals were variable.

**Figure 2.**
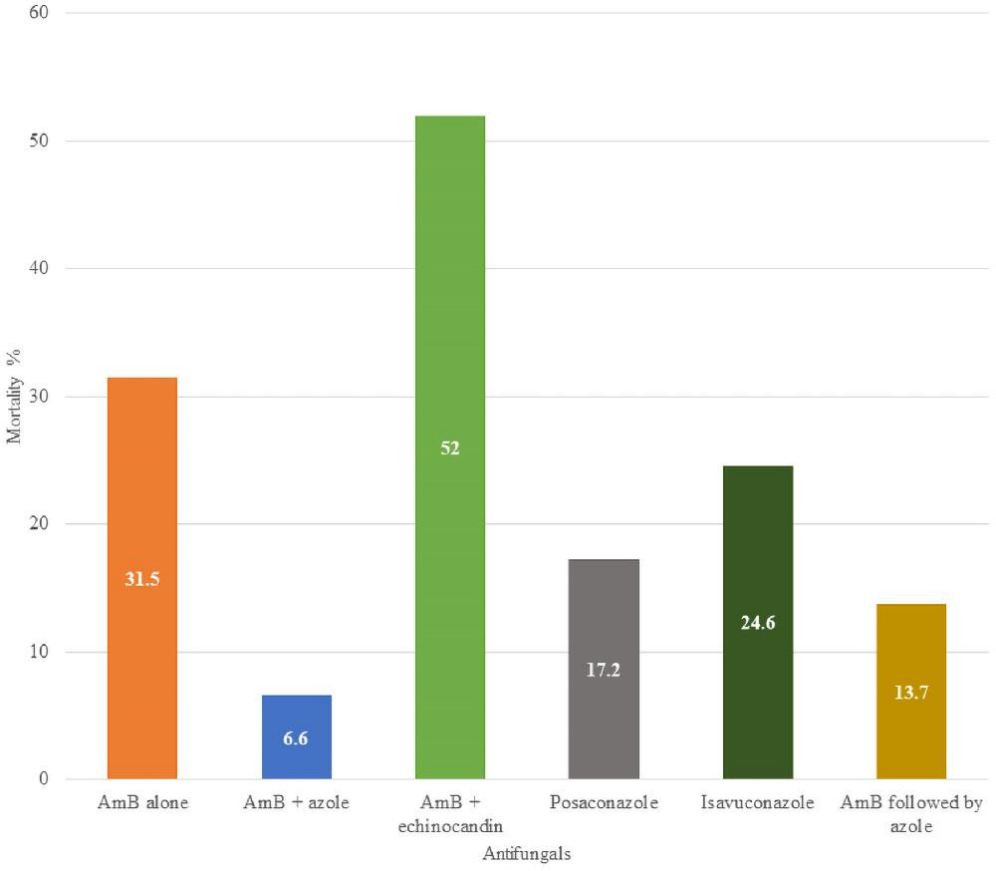
Mortality related to different antifungal therapies and combinations. Significance determined by chi square compared to amphotericin B alone. Abbreviation: AmB, amphotericin B (any formulation).

Mortality despite antifungal therapy was evaluated and compared with the outcome for 74 patients who did not receive an antifungal (table 2, Figure 2). The reasons for lack of treatment were primarily because the diagnosis was established after death.

**Table 2.** Mortality Related to Different Antifungal Strategy and Antifungal Agent; Significance Determined by Chi-Square Compared With Amphotericin B Alone

Antifungal Used	Patients Treated	Patients who Died	Percentage Mortality	*P* Value
Amphotericin B	3749	1181	31.5	-
Amphotericin B + azole	843	56	6.6	<.0001
Amphotericin B followed by azole	357	49	13.7	<.0001
Amphotericin B + echinocandin	25	13	52	.028
Posaconazole	250	43	17.2	<.0001
Isavuconazole	65	16	24.6	.24
Echinocandin	1	1	100	-
No antifungal therapy	74	74	100	<.0001

Among 5290 patients who were treated with antifungals, 1359 died (25.7%). All 74 patients who did not receive antifungal therapy succumbed to illness. Using the overall mortality of amphotericin B (31.5%) as the baseline, posaconazole mortality (17.2%) was significantly lower(*P* < .0001), whereas isavuconazole (24.6%) was not (*P* = .24). Likewise, amphotericin B combined with azoles (6.6%) had significantly lower mortality (*P* < .0001), whereas amphotericin B combined with echinocandins (52%) was worse (*P* = .028). Sequential therapy of amphotericin B followed by azole had much lower mortality (13.7%) (*P* < .0001). All of these comparisons are very tentative given that the dose and duration of antifungals varied widely, timing and extent of surgical therapy also varied, and all the data are retrospective.

#### Surgical Management

**Figure 3.**
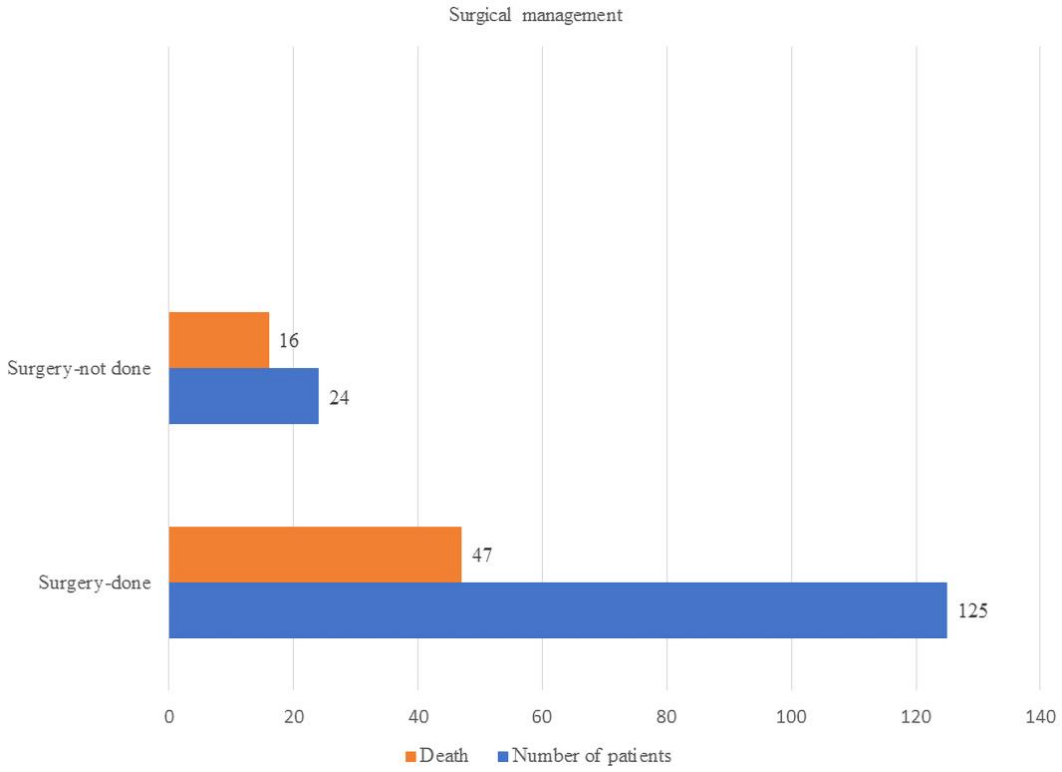
Outcome of surgical management.

We found that 2874 of 5214 (55%) patients (described as a group in 23 articles) underwent surgical management, and overall 1137 of 5214 (21.8%) succumbed to the disease. Unfortunately, the outcome was not clearly given in relation to surgical management in these papers. From 11 publications that provided satisfactory data on surgical management and outcome, 125 of 149 patients underwent surgical resection and 47 of them died (37.6%), compared with 16 (66.7%) of 24 patients who did not undergo surgery (*P* = .008) (Figure 3). Three patients of these 149 patients did not receive antifungal therapy, and all died. The mortality of patients who were operated on and given antifungal therapy were amphotericin B along with surgery 87 (37 deaths), amphotericin B followed by azole 29 (6 deaths), amphotericin B + azole 2 (1 death), amphotericin B + echinocandin 3 (1 death), posaconazole 2 (no deaths).

Undergoing surgery or not did not seem to impact the outcome of pulmonary or disseminated mucormycosis (64% vs 60% and 69% vs 55%, respectively) (Supplementary Figure 6). However, in the small number who were assessable, survival chances were greatly enhanced by surgery for gastrointestinal mucormycosis (0% vs 100%), renal mucormycosis (38% vs 100%), and ROCM (27% vs 80%).

## DISCUSSION

Mucormycosis is a relatively uncommon but serious opportunistic fungal infection. Timely diagnosis prompted by a high index of suspicion is vital for patient survival. We believe this systematic review will contribute to the global epidemiology of mucormycosis, adding some understanding of risk factors, diagnostic methods, and management options.

We observed that the majority of the patients were reported from Asia, and India published most of the Asian cases. Relatively low case numbers were observed in Europe, Middle Eastern countries, and Australia. It is reported that India has a prevalence of mucormycosis that is 80 times that of developed countries [7]. However, the reason for the variation in mucormycosis incidence in these regions is not fully understood. A review reported the most cases from Europe (34%) compared with 31% of cases from Asia from 2000 to 2017 [8], probably due to underreporting of cases in Asian countries during that period.

Our study demonstrated a higher prevalence of mucormycosis among males, in line with other studies [9–11]. However, the reason for this observation is elusive. This could be attributed to the high degree of outdoor activities performed by males compared with females [11]. Whether estrogen plays a protective role against mucormycosis is yet to be explained [12]. The same observation about invasive aspergillosis remains unexplained.

Previous antifungal prophylaxis has been reported in series of mucormycosis cases [8]. This has been more frequently observed in European countries [8]. Even though the time scale and dose of prophylaxis were not reported consistently, 9.6% of patients had a history of previous usage of antifungal prophylaxis. Mucorales fungi are not susceptible to voriconazole [4], and breakthrough mucormycosis has been regularly observed among patients on voriconazole prophylaxis [4]. A systematic review of 851 cases of mucormycosis from 2000 to 2017 reported that the percentage of prior voriconazole usage was 52% [8]. Perhaps surprisingly, breakthroughs were also seen with amphotericin B and posaconazole, given that most strains are susceptible to these antifungal agents, possibly linked to severe immunosuppression or low drug exposure.

Many risk factors are associated with mucormycosis: diabetes mellitus, iron overload, hematopoietic stem cell transplantation, immunosuppressive therapy including corticosteroids, chemotherapy for hematological malignancy, peritoneal dialysis, and extensive skin injury [2]. These predisposing factors differ from region to region and from patient to patient. Skiada reported that diabetes mellitus was the most frequent underlying condition among Indian, Iranian, and Mexican patients [3], while it was hematological malignancy in European patients [6]. Prakash and colleagues also reported a link between uncontrolled diabetes mellitus and mucormycosis among the Indian patient population, where hematological malignancy and solid organ transplantation are proportionately less important [2].

Diabetes mellitus is a well-recognized major risk factor for mucormycosis [6], often in those with ketoacidosis. Diabetes was seen in 76.9% of Asian patients, especially in India (74%). Jeong et al. (2019) in their systematic review also showed that the most common underlying condition was diabetes mellitus (40%) and more frequently reported patients from Asian and African countries compared with Western countries [8]. Annually, 195 000 cases of diabetes-related mucormycosis in India have been estimated, assuming that 55% of all cases are related to poor diabetic control [13]. In this review, only 4.5% of patients with diabetes presented with ketoacidosis.

Both hematological malignancies and hematopoietic stem cell transplantation (HSCT) are frequently linked with mucormycosis cases in the United States, Europe, and Australia [6]. Jeong et al. reported that hematological malignancy (32%) and solid organ transplantation (14%) were the second and third underlying conditions associated with mucormycosis, respectively [8]. According to our analysis, North America has the greatest number of such cases (n = 742, 43%), followed by Europe (n = 557, 32.8%), Australia (n = 29, 48%), and Asia (2.29%). HSCT is also infrequently reported in patients with mucormycosis in developing countries, South American countries (2%), Iran (2%), and India (1%) [6].

Patients on corticosteroids are vulnerable to mucormycosis. Cumulative doses of >600 mg of prednisone increase the risk of cancer patients developing zygomycosis [14]. Patients after pancreas-kidney or liver transplantation who received 2–7 g of methylprednisone have been reported to have mucormycosis [15]. A systematic review reported that corticosteroids predispose to mucormycosis, and 33% of patients were on steroids at the time of presentation [8]. Of the Asian mucormycosis patients, 60.9% had a history of steroid exposure, representing 86.5% of all mucormycosis cases linked with steroids. In contrast, only 24%, 19.3%, and 16.9% of mucormycosis patients from Australia, Europe, and North America had been exposed to steroids. Corticosteroids also increase the risk of death by 419% if continued after the diagnosis is made, whether linked to COVID-19 or not [16]. We were not able to dissect out this important parameter for outcome from the papers we reviewed.

Oxygen therapy was reported in 33% of Indian patients with mucormycosis as a possible risk factor. Only in this population was this reported, which probably reflects the COVID-19 outbreak. It remains uncertain if oxygen contributes directly to mucormycosis risk.

A remarkable steady growth of COVID-19-associated mucormycosis (CAM) was observed during the pandemic [4]. Most of the CAM was reported from India (n = 1614) and comprised 26.4% of all mucormycosis patients reported in Asia. However, they represent 93.1% of CAM in this review, and the rest were shared by the Middle East (4.5%) and Europe (2.3%). Seventy percent of the CAM patients were male, and most cases were ROCM. Severe COVID-19-induced hyperferritinemia, altered glucose homeostasis, and lavish use of corticosteroids for the management of COVID-19 are thought to be potential risk factors for CAM [4]. The estimated mortality rate of CAM lies between 28% and 52% [4]. However, it was not possible to analyze the death rate among CAM patients alone in this review because of the unavailability of specific outcome data.

A few immunocompetent people without trauma or burns develop mucormycosis [2]. In this review, we found that 222 (2.14%) immunocompetent individuals without known predisposing factors had mucormycosis. Jeong et al. reported that almost 50% were not on any immunosuppressive therapy including monoclonal antibodies or chemotherapy [8].

Our review identified 70 patients with renal mucormycosis, almost all reported from India (n = 65) and most among immunocompetent individuals.

The majority of patients in this review were diagnosed by direct microscopy, especially as samples from the nose (and skin or burn/trauma wounds) are readily accessible. Mucorales hyphae emerge as broad, pauci or nonseptate, 90° branching hyphae [4]. The characteristic nature of fungal filaments of Mucorales allows presumptive identification in clinical specimens [17]. Optical brighteners such as Blankophor or Calcofluor white facilitate recognition of the typical broad, sparsely septate, ribbon-like fungal hyphae of Mucorales [18].

Histopathology was the second most common means of establishing the diagnosis of mucormycosis. Although the distinction of Mucorales hyphae from other fungal hyphae is not always reliable, tissue inflammation and infarction certainly differentiate contaminants from true pathogens [6, 17]. Immunofluorescence on tissue was used infrequently, and probably with noncommercial reagents.

Culture claims third place for diagnosis in this review, although fungal blood cultures are almost always negative [19]. Despite their ability to grow fast, only about 50% of cultures yield positive results [6, 19]. This low yield can be attributed to the destruction of delicate viable fungi during the homogenization of specimens, antifungal therapy against Mucorales, or the occurrence of genera that need special conditions [6]. Expertise is required to morphologically identify Mucorales cultures at the species level [17]. Molecular identification of cultured Mucorales species is convenient and remains the gold standard [6]. Matrix-assisted laser desorption ionization–time of flight mass spectrometry (MALDI- TOF-MS) provides protein fingerprints of fungi, and this method allows rapid and less complicated identification [20–22].

Both beta-D-glucan and galactomannan assays cannot be used for the diagnosis of invasive mucormycosis due to lack of those antigens in Mucorales cell walls [23]. Quantitative polymerase chain reaction (PCR) on blood or serum can be included in the diagnosis of mucormycosis, and commercial quantitative PCR assays for Mucorales are now available [24]. Based on the MODIMUCOR multicenter study of 232 patients suspected of having invasive mold disease, serum Mucorales quantitative PCR (qPCR) became positive 4 days before histopathological or mycological results [25]. They reported an acceptable performance of serum Mucorales qPCR, with a sensitivity and specificity of 85.2% and 89.8%, respectively [25].

Antifungal therapy reduces mortality substantially—from 100% to 27.5% overall in this analysis. The most commonly used antifungal was amphotericin B. The death rate of patients who were treated with amphotericin B was 31.5%. Preference toward amphotericin B in the management of mucormycosis may be historically biased [26]. Guinea et al. described liposomal amphotericin B as the most frequent prescription (90%) in their Spanish study of proven/probable mucormycosis [27]. Patel and colleagues observed the use of amphotericin B as a first-line treatment among 81.9% of 465 patients with mucormycosis in India [28]. Stemler et al. reported amphotericin B being used in 92.3% of 310 cases [29]. However, the necessary duration of amphotericin B antifungal treatment is poorly defined and usually tailored to the patient's clinical picture [26].

Our data suggest that the combination of amphotericin B and azole was beneficial, with an overall 6.6% mortality. Given the general concern about antagonism of amphotericin B and azoles, this is reassuring, but not sufficient to recommend dual therapy for all patients, given likely biases in the data. In vitro synergistic activity has been observed in the combination of amphotericin B and posaconazole [30, 31]. The reported clinical success of combined antifungal therapy ranges between 56% and 70% [32], not as high as in our review, perhaps because these were predominantly hematological malignancy patients.

Posaconazole and isavuconazole are the only available oral agents active against Mucorales [33]. Both these azoles have been used for salvage treatment when the use of amphotericin B is not possible or contraindicated [18, 33]. Posaconazole (n = 250) and isavuconazole (n = 65) were used as monotherapy in some studies, with corresponding mortality rates of 17.2% and 24.6%, respectively. Some of these cases arose because of the shortage of amphotericin B in India during the height of the CAM outbreak. One such study of 28 consecutive patients with CAM found 16 (57.1%) cures and 5 (17.9%) improved patients who received either posaconazole or isavuconazole as sole or predominant therapy [34].

A case-matched study of proven or probable mucormycosis from the FungiScope Registry surmised that posaconazole is an alternative therapeutic option for mucormycosis, especially in those with renal dysfunction [35]. A multicenter retrospective study in Spain found that the clinical response to posaconazole for mucormycosis was 55.5% at 3 and 12 months [36].

The US Food and Drug Administration licensed isavuconazole for the treatment of invasive mucormycosis in adult patients in 2015 [37], based on 37 patients in a phase III single-arm open-label trial treated for a median of 84 days [38]. A study of 4 pediatric patients with refractory mucormycosis found complete clinical recovery along with radiologic and mycological clearance after salvage therapy with isavuconazole [39].

Amphotericin B followed by azole was observed among 357 patients, and the death rate among this group was 13.7%. Induction therapy with amphotericin B followed by de-escalation with posaconazole has been recommended for treatment [40], but the focus of the 2019 global guideline for mucormycosis was on substitution of initial amphotericin B with posaconazole or isavuconazole or combination therapy in failing patients, rather than follow on oral therapy [18].

The combination of amphotericin B and echinocandin was seen in 25 patients with a 52% mortality. This small and probably biased experience toward very ill patients also should not govern future practice. Combined antifungal therapy may be more frequent in highly aggressive and extensive forms of mucormycosis [29]. Some positive experiences have been reported in small numbers of patients [41, 42].

Surgical resection of necrotic tissue along with antifungal treatment is recommended to enhance patient outcomes in the management of mucormycosis [18]. Only a few studies provided satisfactory data on surgical management. Deaths were less frequent among the patients who underwent surgical management compared with patients who did not undergo surgery (66.7% vs 37.6%). Combined medical and surgical management is associated with improved survival outcomes compared with exclusive medical management [43]. For example, Pai et al. reported improved survival of 70% of ROCM by combined surgical and antifungal therapy compared with antifungal therapy alone (61%) or surgical management alone (57%) [4]. As long ago as 1994, Tedder and colleagues reported an 11% mortality in pulmonary mucormycosis patients treated surgically compared with 68% mortality in those only treated medically (*P* = .0004) [44]. Their finding was not replicated in our data. However, surgical treatment is not free of complications. A retrospective observational study evaluating surgical complications of 146 patients with acute invasive fungal rhinosinusitis, most with mucormycosis, showed that bleeding is the most common intraoperative complication, followed by cerebrospinal fluid leak, orbital hematoma, nasolacrimal duct trauma, and periorbital hematoma [45]. A number of postsurgical complications including synechiae, hypoesthesia, decreased vision, facial pain, facial deformity, diplopia, headache, anosmia, dental pain, earache, hyposmia, and periorbital ecchymosis have been documented [43].

Key limitations of our work relate to the lack of detail and complexity of most patients in the literature. Organ-specific details are sometimes difficult to obtain because studies often report clinical forms collectively, as other reviewers also found [43]. Unfortunately, some of the articles failed to provide sufficient information on patient management. Both deoxycholate and lipid amphotericin B formulations were used, but many articles did not provide information on formulation, dose, or duration. Outcomes were often aggregated, so we have fewer data points for the outcomes analysis, especially with regard to surgery. In addition, surgical resections vary in timing, how many are done in individual patients, and whether supported by frozen section biopsies to guide margins, as well as other factors.

Overall, this extensive literature review sets a baseline for therapeutic outcomes of mucormycosis for any future prospective studies. An antifungal therapy with an active agent is essential for survival, further enhanced if surgical resection accompanies it. Both combination and sequential therapy of posaconazole or isavuconazole with or after amphotericin B seem to be superior strategies, but this could reflect survivor bias. Posaconazole and isavuconazole as monotherapy also have promise as alternatives to amphotericin B. Heightened awareness of mucormycosis in India during COVID-19 possibly allowed for earlier diagnosis and better outcomes overall, but this remains conjecture.

## Supplementary Material

ofad704_Supplementary_Data

## Supplementary Data

Supplementary materials are available at *Open Forum Infectious Diseases* online. Consisting of data provided by the authors to benefit the reader, the posted materials are not copyedited and are the sole responsibility of the authors, so questions or comments should be addressed to the corresponding author.
