# Supplementary material for: A Systematic Review of the Therapeutic Outcome of Mucormycosis
Source: Open Forum Infect Dis. 2023 Dec 30;11(1):ofad704. doi: 10.1093/ofid/ofad704 (PMC10823420; doi:10.1093/ofid/ofad704)

Sigera and Denning

A systematic review of the therapeutic outcome of Mucormycosis

Supplementary appendix

Figure S1: Flow diagram of publication screening and selection. (LAB = laboratory-based studies, LCR = Case series with <10 cases, RW = Literature review, SRW = Systematic review).

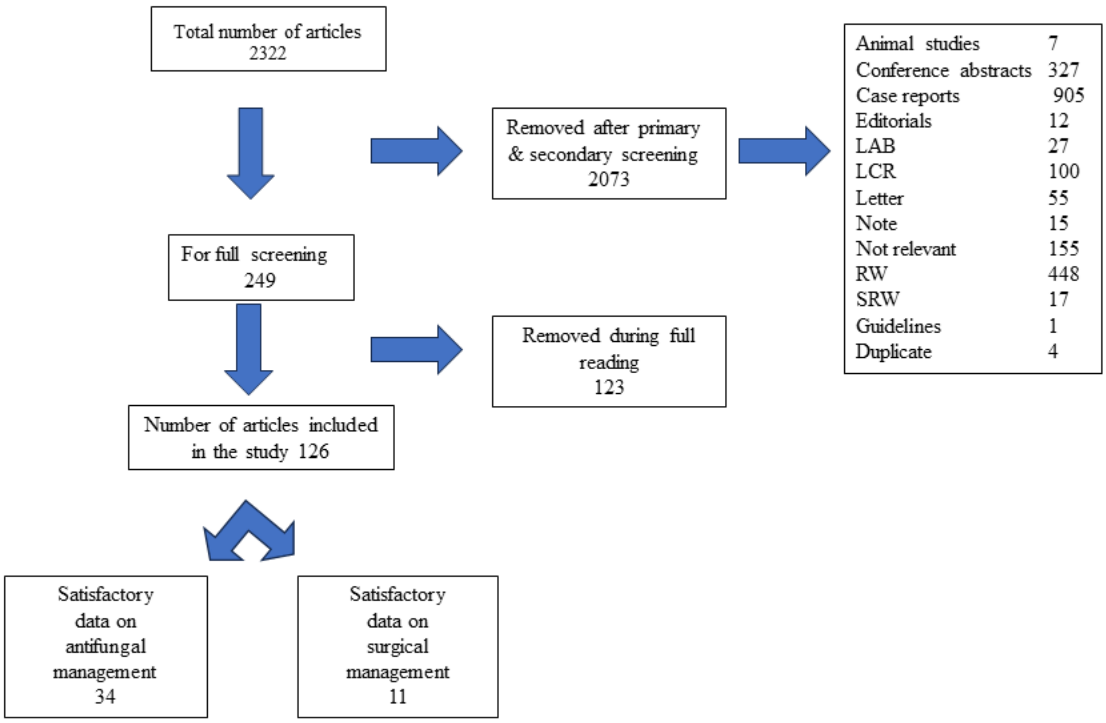

Figure S2: The regional distribution of mucormycosis patients.

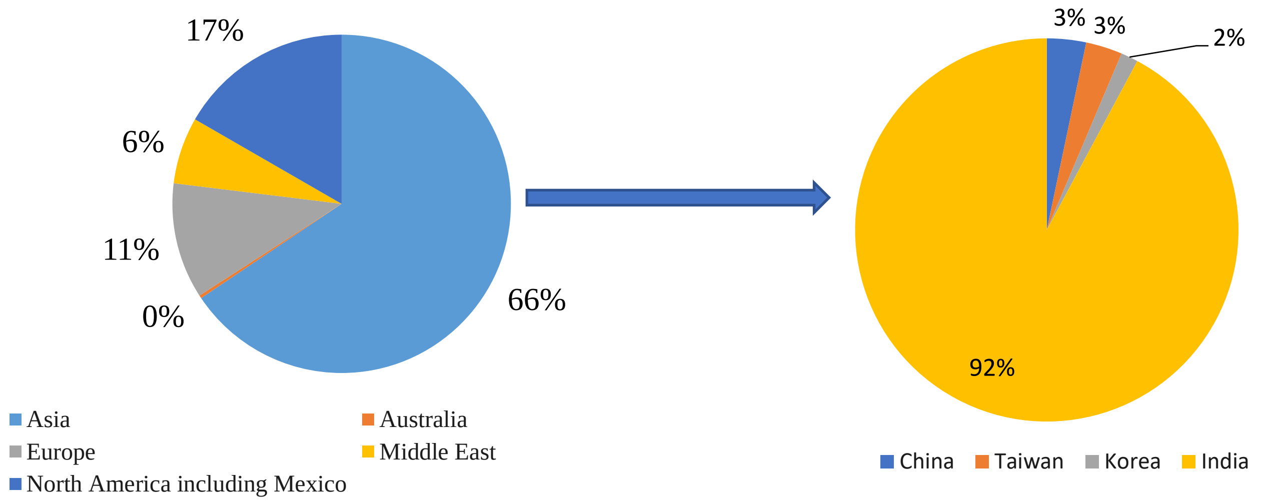

Figure S3: Use of antifungal prophylaxis prior to onset of mucormycosis

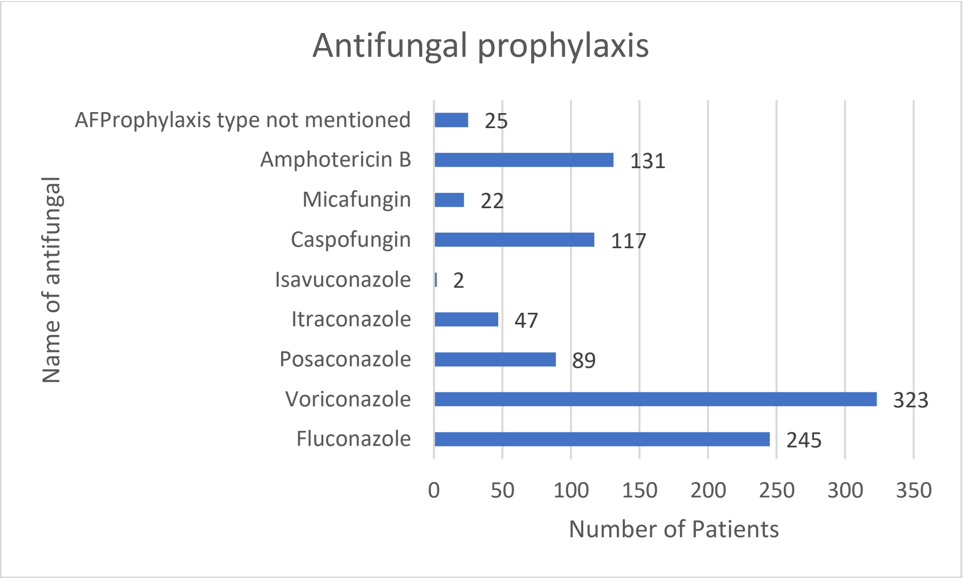

Figure S4: Clinical form of mucormycosis (ROCM = rhino-orbito-cerebral mucormycosis)

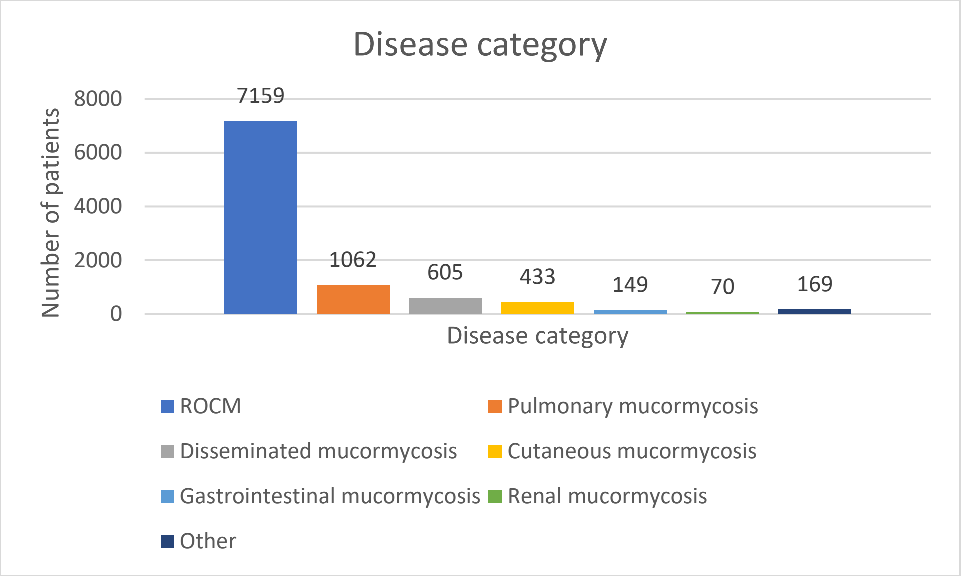

Figure S5: Antifungals used in the management

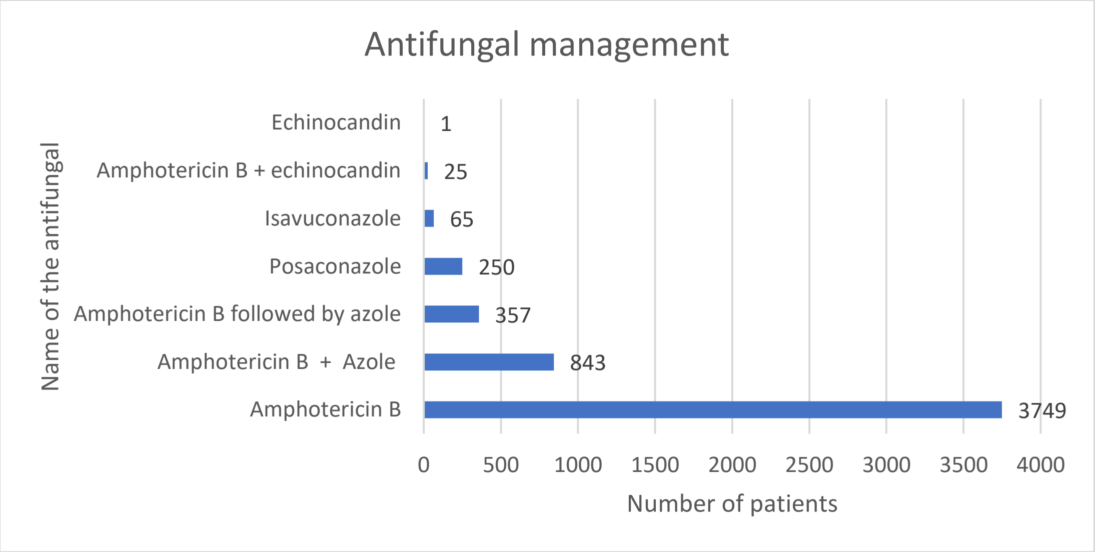

Figure S6: Comparison of surgical management of different clinical forms.

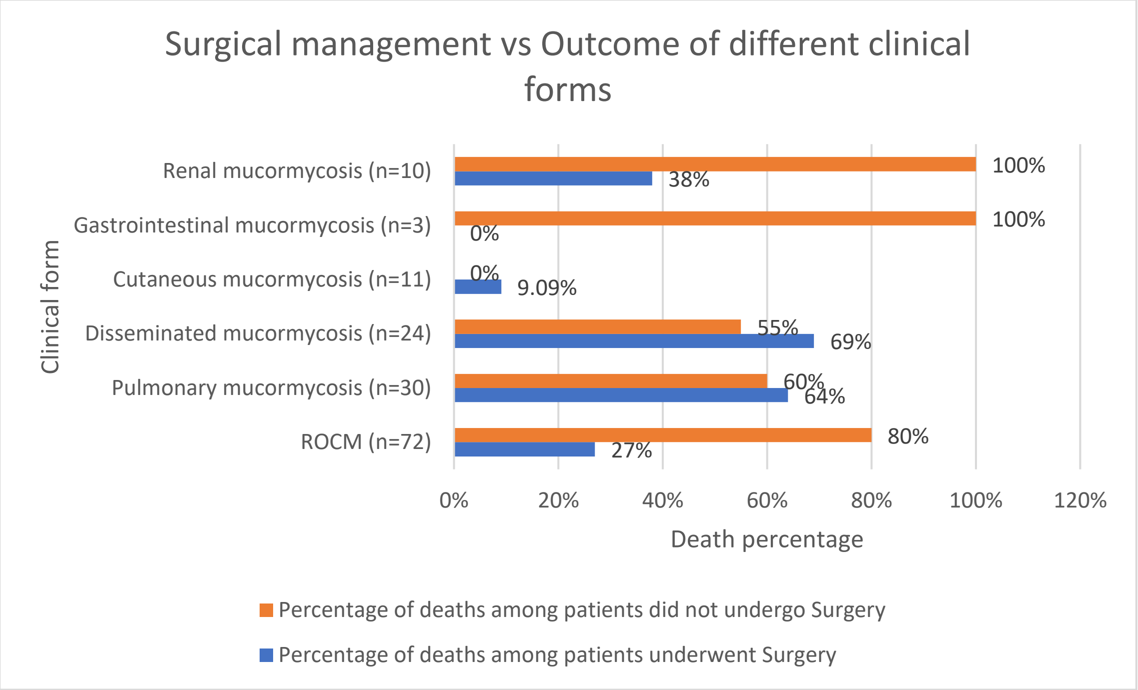

Supplement: ofad704_Supplementary_Data [file ofad704_supplementary_data.zip › SIgera supplementary appendix v2.pdf]
