# Supplementary material for: A Systematic Review of the Therapeutic Outcome of Mucormycosis
Source: Open Forum Infect Dis. 2023 Dec 30;11(1):ofad704. doi: 10.1093/ofid/ofad704 (PMC10823420; doi:10.1093/ofid/ofad704)
Supplement: ofad704_Supplementary_Data [file ofad704_supplementary_data.zip › SIgera supplementary appendix v2 (1) (1).docx]

**Sigera and Denning**

# A systematic review of the therapeutic outcome of Mucormycosis

**Supplementary appendix**

**Figure S1: Flow diagram of publication screening and selection. (LAB = laboratory-based studies, LCR = Case series with <10 cases, RW = Literature review, SRW = Systematic review).**

**
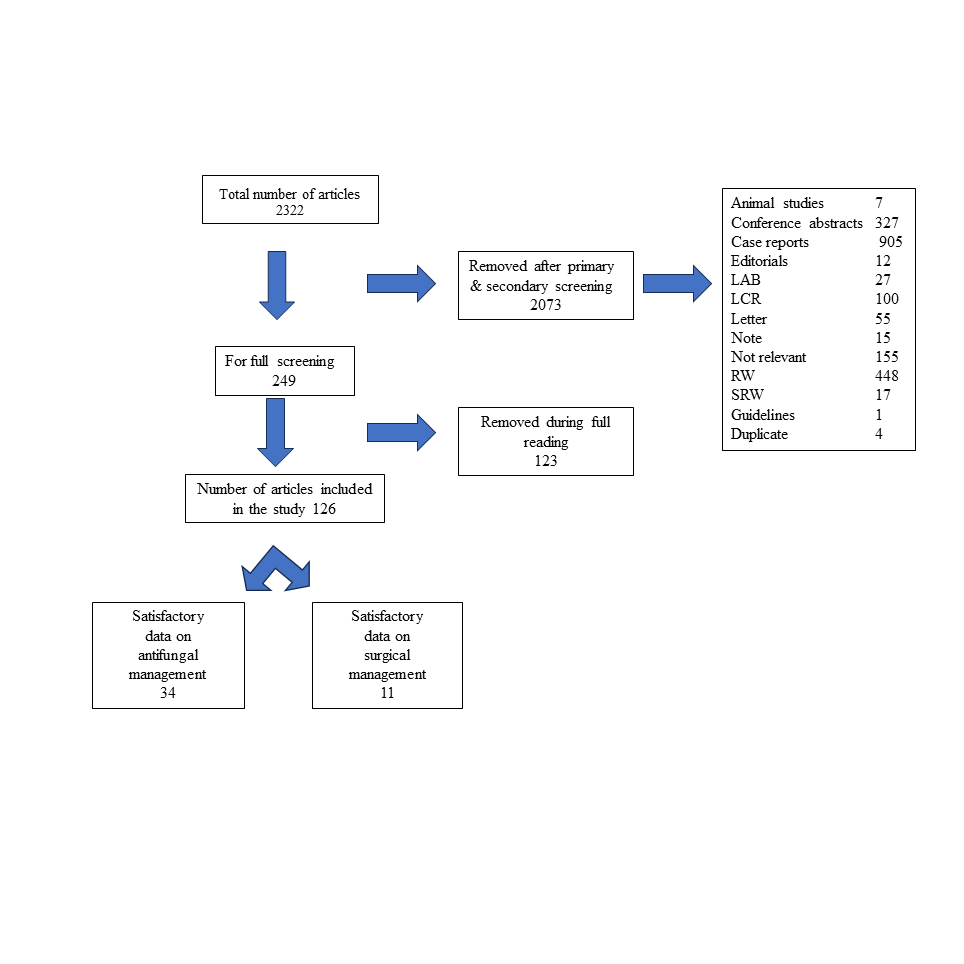
**

**Figure S2: The regional distribution of mucormycosis patients.**

**Figure S3: Use of antifungal prophylaxis prior to onset of mucormycosis**

**Figure S4: Clinical form of mucormycosis (ROCM = rhino-orbito-cerebral mucormycosis)**

**Figure S5: Antifungals used in the management**

**Figure S6: Comparison of surgical management of different clinical forms.**
